# Progress in Dermatology

**Published:** 1903-09-12

**Authors:** 


### Progress in Dermatology.

**Leprosy.**—Mr. Jonathan Hutchinson[1] has returned from India with his views strengthened with regard to the part played by fish in the conveyance of leprosy. He can find no class, with the one exception of the Jains, who by-the-bye do not furnish cases of leprosy, which excludes fish from the dietary. He was much struck by the tabulated figures with regard to some "Salsette Christians" in an asylum near Bombay. These are Roman Catholic Christians from the island of Salsette, and mainly a race of fishermen, who furnish leprous patients very much out of their proportion, compared with that of the Jains, Hindus, and Mussulmans. He finds in this an indication of the important part which a fish diet —the diet habitual to them owing to their trade and religion—plays in the ætiology of this disease. He can find no evidence of the hereditariness, nor of the contagiousness of the disease. Leprosy was almost wholly absent from Cape Colony, Natal, and the Sandwich Islands until factories for the salting of fish were instituted. Hutchinson holds that the danger lies in the ingestion of decomposing fish.[2] In answer to those who have pointed out that peoples who cannot easily obtain fish show the disease, while those who have easy access to rivers, etc., do not show it, he holds that the fact that fish is not to be easily obtained fresh makes it more possible that when they do get it it may be decomposing. Finucane, in commenting, stated that in Fiji leprosy was largely due to the insanitary surroundings. Hancock, of Assam, and Van Dam, of British Guiana, both state that fish is largely eaten by the people: in British Guiana rotten fish is largely consumed. Tonkin,[3] whose extensive experience in the Central Sudan has given him special facilities for the investigation of leprosy in these parts, deals exhaustively with the question of possible heredity in 220 cases which he has thoroughly investigated and tabulated. His figures and arguments would seem conclusive that parental transmission of this disease does not exist, but that early leprous environment tends to earlier development of the disease in children. Against the hereditary theory he urges that as leprosy tends to interfere with the sexual functions the tendency would be for the disease to die out altogether. He states also that many instances of recovery from most grades of leprosy do actually occur, and that this is to him a matter beyond debate. He considers that a period of 15–20 years completely covers the actual morbid processes; so that if a patient has survived the disease onset by 20 years it is safe to assume that he has survived the disease itself. In the matter of diet he points out that animal food is eaten very sparingly by the population of the Sudan, but the meat is frequently not fresh, and that fish is not abundant and is considered more of a delicacy even than meat. He looks for a common factor in all countries where leprosy is found, and finds one in the insanitary condition of clothes and bedding frequently never washed at all and shared communistically, and another he finds in a diet largely consisting of carbohydrates, in which these elements are represented to an extent which disturbs the proper proportion of the nitrogenous. This inefficiency of diet he holds not of course to be the cause of the disease, but one which assists to determine its incidence. Van Hontum[4] reports that he has cultivated the B. lepræ on fish broth, subcultures being made with difficulty on glycerine beef agar. This bacillus is not the same as that of Hansen. It decolorizes with Ziehl-Neelsen stain and is somewhat thicker. There are no satisfactory results in agglutination experiments. Bacteriolysis occurred, however, in leper serum in dilution of 1 in 50 to 1 in 100 (Pfeiffer's reaction), which was not the case with other sera. The treatment of leprosy is undertaken hopefully by Razlag,[5] who has cured three cases and has a fourth still under treatment, but greatly improved, it having been a very bad case. There is nothing specific in the treatment advocated. It consists largely in open-air life, cleanliness, and the use of baths. These latter are cold or warm, of fresh, salt, or medicated water, according to the indications of the case. Iodine, tannic acid, and pot. permanganate being the drugs used. Wounds are dressed with zinc chloride or sulphate, hydrogen peroxide, ichthyol, chrysarobin, arsenic, iodine, creasote, croton oil, or salicylic acid. Iodoform he never uses. He uses friction to the skin, massage being made with croton or Chaulmoogra oils. Leeches are judiciously applied for œdema. Internally jaborandi, strong tea or coffee as sudorifics: arsenic, strychnine, ichthyol, sodium salicylate, and creasote. He warns against allowing the wounds to heal up too rapidly, as this prolongs the nodules. The time taken in the treatment is a year or more in favourable cases.

[1] Brit. Med. Jour., May 23. [2] Ibid., May 30. [3] Lancet, April 18. [4] Brit. Med. Jour., Feb. 14. [5] Ibid., May 16.

(*To be continued.*)

